# Targeting cell death in NAFLD: mechanisms and targeted therapies

**DOI:** 10.1038/s41420-024-02168-z

**Published:** 2024-09-07

**Authors:** Hui-li Xu, Sheng-rong Wan, Ying An, Qi Wu, Yi-hang Xing, Chen-hao Deng, Ping-ping Zhang, Yang Long, Bu-tuo Xu, Zong-zhe Jiang

**Affiliations:** 1 Department of Endocrinology and Metabolism, The Affiliated Hospital of Southwest Medical University, Luzhou, Sichuan PR China; 2 Metabolic Vascular Disease Key Laboratory of Sichuan Province, Luzhou, Sichuan PR China; 3 Sichuan Clinical Research Center for Nephropathy, Luzhou, Sichuan PR China; 4 Department of Pathology, The Affiliated Hospital of Southwest Medical University, Luzhou, Sichuan PR China; 5 Precision Pathology Diagnosis for Serious Diseases Key Laboratory of LuZhou, Luzhou, Sichuan PR China; 6 The People’s Hospital of Pingyang, Wenzhou, Zhejiang PR China; 7 Academician (Expert) Workstation of Sichuan Province, The Affiliated Hospital of Southwest Medical University, Luzhou, Sichuan PR China

**Keywords:** Inflammasome, Metabolic syndrome, Oncogenesis

## Abstract

Nonalcoholic fatty liver disease (NAFLD) is a group of chronic liver disease which ranges from simple steatosis (NAFL) to non-alcoholic steatohepatitis (NASH) and is characterized by lipid accumulation, inflammation activation, fibrosis, and cell death. To date, a number of preclinical studies or clinical trials associated with therapies targeting fatty acid metabolism, inflammatory factors and liver fibrosis are performed to develop effective drugs for NAFLD/NASH. However, few therapies are cell death signaling-targeted even though the various cell death modes are present throughout the progression of NAFLD/NASH. Here we summarize the four types of cell death including apoptosis, necroptosis, pyroptosis, and ferroptosis in the NAFLD and the underlying molecular mechanisms by which the pathogenic factors such as free fatty acid and LPS induce cell death in the pathogenesis of NAFLD. In addition, we also review the effects of cell death-targeted therapies on NAFLD. In summary, our review provides comprehensive insight into the roles of various cell death modes in the progression of NAFLD, which we hope will open new avenues for therapeutic intervention.

## Highlights

- Hepatocyte death is a critical event in the progression of NAFLD.
- Different types of hepatocyte death participate in the occurrence and development of NAFLD, including apoptosis, necroptosis, pyroptosis and ferroptosis.
- Drugs or compounds targeting the hepatocyte death signaling pathway is a potential direction for the treatment of NAFLD and needs to be further studied.

## Facts

- The incidence of NAFLD is increasing worldwide and it has become a major chronic liver disease that poses a significant threat to public health.
- Several forms of programmed cell death, including apoptosis, necroptosis, pyroptosis, and ferroptosis, play an integral role in the pathogenesis of NAFLD.
- A growing body of evidence suggests that various cell death modes may serve as a promising target for the prevention and treatment of NAFLD.

## Open questions

- Which type of cell death mode plays a dominant role in NAFLD?
- Are there other types of cell death modes such as cuproptosis that are also involved in the development of NAFLD?
- Given that various cell death modes occur in different types of cells during the pathogenesis of NAFLD, what are the underlying links among them?

## Introduction

NAFLD is defined as a clinicopathological syndrome that is not due to alcohol and other well-defined liver-damaging factors. The incidence of NAFLD is increasing worldwide with the rapid economic development and lifestyle changes and it has become a major chronic liver disease that poses a significant threat to public health [1]. Depending on the severity of the disease, NAFLD includes non-alcoholic fatty liver (NAFL), non-alcoholic steatohepatitis (NASH), liver fibrosis, and liver cirrhosis. The most widely accepted pathogenesis of NAFLD/NASH is the current “multiple whammy” theory, which encompasses various factors such as genetic susceptibility, epigenetics, intrahepatocellular metabolic and signaling pathways, cellular interactions within the liver, and hepatocyte injury and death, and sheds light on the heterogeneity of NAFLD [2, 3]. The primary causative factors of NAFLD are predominantly attributed to the excessive accumulation of fatty acids in hepatocytes. This leads to lipotoxicity, oxidative stress, organelle dysfunction, and inflammatory responses, which subsequently initiate a cascade of cell death in the liver including apoptosis, necroptosis, pyroptosis, and ferroptosis, and ultimately exacerbate NAFLD progression. Hepatocyte death is a critical event in the progression of NAFLD, therefore a better characterization of hepatocyte death in NAFLD may lead to novel therapeutic.

Since the classification of cell death subroutines was updated by the Nomenclature Committee on Cell Death in 2018, cell death can be divided into accidental cell death (ACD) and regulated cell death (RCD) based on perspectives of morphology, biochemistry, and function. ACD is a process that cells exposed to very harsh environmental conditions (e.g., severe physical, chemical, or mechanical injury) disassemble in a virtually instantaneous and uncontrollable manner. RCD, also known as programmed cell death (PCD), is initiated and propagated by specific molecular mechanisms and is subject to regulation. It can be divided into several subroutines, such as apoptosis, necroptosis, pyroptosis, and ferroptosis [4, 5]. Based on accumulating evidence, RCD is closely related to the initiation and progression of NAFLD [6, 7]. Our review outlines the molecular mechanisms and processes of four major RCD subroutines related to NAFLD. The focus is on the latest advancements in targeting cell death for NAFLD therapy and the mechanisms of the various NAFLD therapies currently available, which mainly depend on different cell death modalities.

## Different types of cell death in NALFD

### Apoptosis

#### Overview of apoptosis

**Fig. 1:**
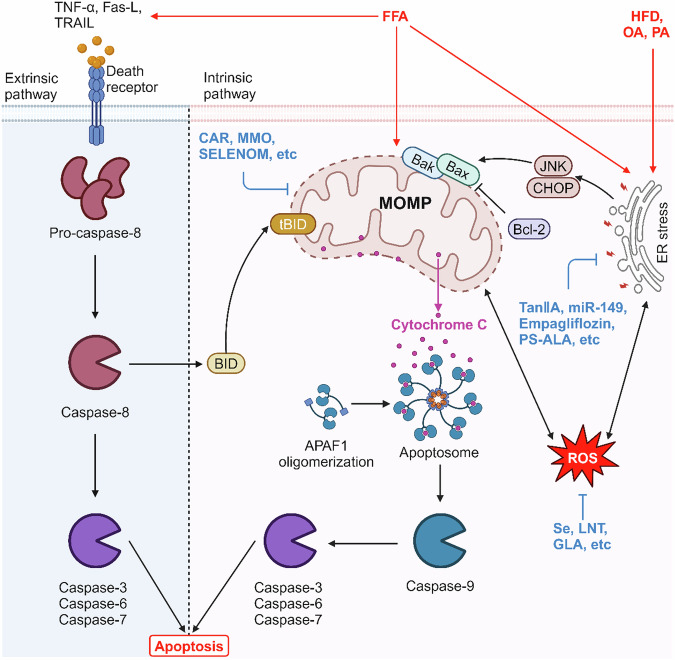
Mechanism of apoptosis in NAFLD. In NAFLD state, HFD, OA, PA, and FFA lead to hepatocyte apoptosis by inducing increased expression of death receptors, disrupting mitochondrial function, and triggering ER stress.

Apoptosis is a genetically determined process to terminates a cell’s life automatically that performs an important role in maintaining organismal homeostasis. It is characterized morphologically by chromatin condensation, cell shrinkage, and the eventual lysis of cells into apoptotic bodies through budding, which is subsequently cleared by phagocytosis. Mechanistically, the apoptotic pathways can be triggered by the exogenous or endogenous pathways [7, 8] (Fig. 1). In detail, the exogenous apoptotic pathway is typically mediated by cell surface death receptors (DRs), such as tumor necrosis factor receptor (TNFR), factor related apoptosis (Fas), TNF-related apoptosis-inducing ligand receptors (TRAIL-R), or toll-like receptors (TLRs) [7]. When bound to natural ligands, these receptors initiate an intracellular cascade reaction that results in the production of a death complex, which then activates caspase-8, followed by caspase-3/6/7 [7]. In the endogenous apoptotic pathway, various cellular stress, such as nuclear DNA damage, cytokine deprivation, endoplasmic reticulum stress (ERs), lysosomal permeabilization, and oxidative stress generate intracellular death signals to promote mitochondrial dysfunction and subsequently mitochondrial outer membrane pore formation (MOMP). Next, cytochrome c is released from the mitochondria into the cytoplasm through the mitochondrial membrane gap and binds to apoptosis protein activator-1 (Apaf-1) in the cytoplasm. And then, Apaf-1 undergoes oligomerization and activates caspase-9 to constitute an activation complex. This complex further activates downstream effector caspase-3/6/7, ultimately leading to the execution of apoptosis [8]. In the context of apoptosis, the mitochondrial pathway plays a crucial role in promoting caspase-8-dependent apoptotic signaling. Specifically, caspase-8 is involved in the cleavage of Bid, a death agonist with a BH3-interacting structural domain. This cleavage generates a truncated form of Bid, named tBid, which translocates to the mitochondria and disrupts the mitochondrial outer membrane. This disruption leads to MOMP, thereby integrating the two pathways into a final common pathway [9]. The regulation of MOMP is governed by the Bcl-2 protein family, comprising anti-apoptotic members (Bcl-2, Bcl-xL) and pro-apoptotic members (Bax, Bak). ERs can induce apoptosis through c-Jun N-terminal kinase (JNK) and C/EBP homologous protein (CHOP), thereby activating the pro-apoptotic protein Bax and disrupting mitochondrial function [10, 11].

#### Apoptosis in NAFLD

Numerous studies have demonstrated that hepatocyte apoptosis plays a crucial role in the progression of NAFLD. Within the population of NAFLD patients, there is a remarkable increase in the number of TUNEL-positive hepatocytes and the level of caspase-3 activity compared to normal individuals. Various animal models of NAFLD have also shown increased levels of caspase-3/8/9 and decreased cytochrome c in mitochondria. Furthermore, the degree of hepatocyte apoptosis is positively correlated with the level of liver inflammation and the stage of fibrosis. Studies have demonstrated pharmacological inhibition or selective knockdown of caspases effectively reduces hepatic apoptosis and inhibits the development of inflammation and fibrosis [7, 12, 13]. All of this indicates that apoptosis is a significant feature for NAFLD [14, 15]. In some clinical studies, the expression of several DRs, including Fas, TRAIL-R1, TRAIL-R2, and TNF receptor 1(TNFR1), was shown to be increased in the livers of NAFLD patients [16–18]. Moreover, the TRAIL knockout mice have been found to avoid diet-induced NASH [19]. In response to free fatty acids (FFA) treatment, cell surface receptors aggregate, leading to caspase-8 recruitment and ultimately resulting in cell death. Notably, compared to the concentrations required for FFA-mediated Fas sensitization or FFA-induced lipid apoptosis, the concentrations required for TRAIL sensitization by FFA are significantly lower. This may be due to the upregulation of DR5 expression through ERs, and then the JNK-dependent mechanisms induced the hepatocyte steatosis sensitivity to TRAIL [20, 21]. In physiological conditions, JNK takes part in cell growth, survival, and death. The FFA induces JNK-dependent hepatocyte apoptosis by activating Bax expression, which triggers the mitochondrial apoptotic pathway [22]. Similarly, oleic acid (OA) treatment upregulated the expression of p-AKT, p-JNK, Bax, and caspase-3 and decreased the Bcl-2 expression in LO2 cells, indicating that OA can induce apoptosis via AKT/JNK signaling in a Bcl-2/ Bax/caspase-dependent manner under stress conditions. This apoptotic mode is associated with mitochondrial dysfunction, as OA treatment causes disruption of mitochondrial membrane potential. Consequently, the impairment of mitochondrial function hinders the process of fatty acid β-oxidation, leading to intracellular fatty acid accumulation which, in turn, stimulates reactive the production of reactive oxygen species (ROS), and excessive ROS further aggravates mitochondrial dysfunction, ultimately triggers apoptosis [23]. In AML-12 cells, the upregulation of Bax, caspase-3, and caspase-9 expressions, as well as the increased apoptosis rate, were observed upon exposure to palmitic acid (PA) [24]. In vivo experiments have demonstrated a significantly increase in caspase-3/9 and Bax expression accompanied by a decrease in the expression of Bcl-2 and cytochrome c in mitochondria within hepatocytes in HFD-fed mice. These findings indicate that fatty acid can indeed promote the development of NAFLD by inducing apoptosis in hepatocytes triggered by mitochondrial dysfunction [15].

Many studies have shown that excessive lipid accumulation in hepatocytes can lead to apoptosis caused by ERs. In HFD-fed mice and OA-treated hepatocytes, the endoplasmic reticulum exhibits significantly swelling and dilation. Additionally, the expression of glucose regulated protein 78 (GRP78) and CHOP, both key hallmarks of ERs, is dramatically enhanced, and the IRE1α/TRAF2/JNK signaling pathway is activated, which in turn increased the expression of Bax and inhibited the expression of Bcl-2, leading to hepatocyte apoptosis [8, 25]. Similarly, PA causes HepG2 cytotoxicity, triggers ERs through the activation of protein kinase R-like ER kinase (PERK)-eukaryotic initiation factor 2α (eIF2α) and activating transcription factor 6 (ATF6) pathways, increases the expression of ERs-associated apoptotic protein CHOP, and ultimately leads to apoptosis [26].

Therefore, in NAFLD, apoptosis appears to be the predominant form of cell death, as there is evidence for the involvement of both pathways of apoptosis. Studying the molecular mechanism of apoptosis can provide a deeper understanding of its association with NAFLD and lead to therapeutic strategies.

#### Apoptosis-targeted therapies in NAFLD

As a result, an increasing number of drugs have been found to alleviate NAFLD progression by targeting apoptosis (Table 1). An increasing number of studies have shown that increased levels of cellular FFA serve as a catalyst for mitochondrial oxidative damage and apoptosis. Some antioxidants, such as Selenium (Se), prevent apoptosis by improving cellular antioxidant defenses, reducing ROS production, and maintaining cellular redox homeostasis [27]. Similarly to Se, VD supplementation can also reduce oxidative stress-related indexes, which further exerts a therapeutic effect on NAFLD. Besides, HFD-induced mice supplemented with active VD showed decreased levels of P53, P21, and P16, which proved that VD inhibited hepatocyte apoptosis by inhibiting the P53 pathway, but its specific mechanism needs to be investigated [28]. Lentinan (LNT) treatment reduced the ratio of Bax/Bcl-2 protein and caspase-3 activity, ameliorated oxidative stress, and reduced lipid deposition in both PA-induced AML-12 cells and HFD-induced mouse livers. Importantly, these benefits were reversed when peroxisome proliferator-activated receptor (PPARα) was knocked down, suggesting that LNT reduces apoptosis and oxidative stress and delays the progression of NAFLD via the PPARα pathway [29]. As a polyunsaturated fatty acid (PUFA), γ-linolenic acid (GLA) not only reduces intracellular lipid deposition in hepatocytes, but also activates autophagy via the LKB1–AMPK–mTOR pathway, which in turn decreases the expression level of Bax/Bcl-2 and reduces apoptosis [24]. Hydroxycitric acid (HCA), a major component of the natural fruit *Garcinia cambogia*, exhibits antioxidant effects and activates the nuclear factor erythroid 2-related factor 2 (NRF2)–antioxidant response element (ARE) pathway. This activation inhibits ROS production, regulates the Bcl-2/Bax ratio and PARP cleavage, and reduces apoptosis, in both HFD-induced and FFA-induced HepG2 cells, thereby delaying the process of NAFLD [30]. In the NAFLD model of zebrafish, we observed that swimming exercise can inhibit excessive ROS production, reduce caspase-3 and Bax expression, and increase Bcl-2 levels by activating recombinant Sirtuin 1 (SIRT1)/AMP-activated protein kinase (AMPK) signaling-mediated NRF2, which in turn reduces oxidative stress and ROS-induced apoptosis in zebrafish liver, decreases lipid accumulation in zebrafish NAFLD model liver, and attenuates liver injury [31].

**Table 1:** Apoptosis-targeted therapies in NAFLD.

Treatments	Model	Targets	Signaling pathways or molecular mechanisms	Ref.
PinX1 knockdown	HFD-induced mice and primary mice hepatocytes	mTERT	Improves mTERT activity	[45]
Se	Primary rat hepatocytes	Oxidative stress	Antioxidant	[27]
Vitamin D	HFD-induced mice	Oxidative stress	Antioxidant	[28]
Exercise	HFD-induced zebrafish	Oxidative stress	Activates SIRT1/AMPK/NRF2 signaling	[31]
	HFD-induced rat and HepG2 cells	Srit1	Activates Sirt1-mediated Drp1 acetylation	[33]
LNT	HFD-induced mice and AML-12 cells	Oxidative stress	Activates PPARα pathway	[29]
GLA	AML-12 cells	Oxidative stress	Activates LKB1–AMPK–mTOR pathway	[24]
HCA	HFD-induced mice and HepG2 cells	Oxidative stress	Activates NRF2–ARE pathway	[30]
PK	HFD-induced rat	Mitochondria	Improves mitochondrial function	[15]
A22	HFD-induced mice and HepG2 cells	Gene promoter i-motif	Upregulates Bcl-2 transcription and translation	[32]
BCATc inhibitor 2	LO2 and HepG2 cells	p-AKT, p-JNK, and mitochondria	Inhibits Bcl-2/Bax/caspase axis and AKT/ERK signaling and improves mitochondrial function	[23]
EGCG	HFD-induced mice and LO2 cells	Mitochondria	Inhibits ROS/MAPK pathway	[34]
Sulforaphane	HFD-induced mice and HepG2 cells	Ceramide	Inhibits the ceramide	[35]
Myriocin	HFD-induced rat	Ceramide	Inhibits the ceramide	[36]
Silicon	HFD and HSHCD-induced rat	Mitochondria	Downregulates the mitochondrial (intrinsic) pathway process	[37]
CAR	HFD-induced mice and AML-12 cells	PRDX3	Improves mitochondrial function	[38]
MMO	Human Chang liver cells	Mitochondria	Improves mitochondrial function	[39]
SELENOM	HFD-induced mice and AML-12 cells	Parkin	Activates AMPKα1–MFN2 pathway and improves mitochondrial function	[40]
Tan IIA	HepG2 cells	ER stress	Inhibits PERK/eIF2α and ATF6 signaling pathway	[26]
Empagliflozin	HFD-induce mice	ER stress	Inhibits ER stress	[41]
MiR-149	HFD-induced mice and primary mice hepatocytes	ER stress	Downregulates the ATF6 signaling pathway	[42]
α-Tocopherol	HCD-induced rabbit	ER stress	Reduces JNK/c-Jun/inflammatory axis and JNK/CHOP/apoptotic signaling	[43]
α-Linolenic acid	HFD-induced mice and HepG2 cells	AMPK	Inhibits IRE1α/TRAF2/JNK signaling pathway	[44]

Emerging evidence indicates that mitochondrial dysfunction plays an important role in the progression of NAFLD, the dysfunctional metabolism associated with NAFLD further exacerbates mitochondrial dysfunction, thereby accelerating the development of NAFLD. However, a large number of drugs have been found to modulate mitochondrial function in a variety of ways, thereby reducing the occurrence of mitochondria-dependent apoptosis in hepatocytes and thus exerting a protective effect against NAFLD. For example, *Polygonatum kingianum* (*PK*) has been shown to improve mitochondrial function by enhancing mitochondrial ROS (MtROS) scavenging, and it reverses the upregulation of caspase-3/9 and Bax expression, while restoring the decreased levels of Bcl-2 and cytochrome c in hepatocytes of HFD-induced NAFLD rats [15]. The acridone derivative A22 has demonstrated the ability to upregulate the Bcl-2 expression and block the caspase cascade reaction and mitochondrial apoptotic pathway by selectively binding and stabilizing the Bcl-2 gene promoter i-motif. Consequently, it reduces hepatocyte apoptosis in NAFLD/NASH models and effectively attenuates liver injury [32]. It is well-accepted that aerobic exercise has shown beneficial effects in the prevention and treatment of NAFLD. In a study by Hu et al. on HFD-fed rats and OA-treated HepG2 cells, aerobic exercise regulated the acetylation and activity of dynamically related protein 1 (Drp1) by activating Sirtuins1 (Srit1). This, in turn, reduces ROS generation in hepatocytes, increases mitochondrial membrane potential, mitigates mitochondrial dysfunction, and thus prevents NAFLD [33]. Researchers have found that supplementation of branched-chain amino acids exacerbates OA-induced lipid accumulation and apoptosis. However, BCATc inhibitor 2, a branched-chain amino acid transaminase inhibitor, attenuates the OA-induced activation of JNK and AKT signaling pathways, as well as Bcl-2/Bax/caspase axis. It also reduces MtROS production and prevents potential disruption of mitochondrial membranes, thereby maintaining mitochondrial function and thus exert a protective effect against NAFLD [23]. Epigallocatechin-3-gallate (EGCG) has the potential to attenuate mitochondria-dependent apoptosis in OA-treated LO2 cells and HFD-induced mice hepatocytes by inhibiting the ROS-mediated mitogen-activated protein kinase (MAPK) pathway [34]. Saturated fatty acids (SFA) inhibited apoptosis and downregulated cleaved caspase-3 both in vitro and in vivo, and decreased ceramide and ROS levels. These findings suggest a potential preventive effect against the development of NAFLD. Specifically, ceramide, acting as a second messenger for FFA-induced apoptotic effects, can be targeted to mitigate NAFLD [35]. For instance, the ceramide synthesis inhibitor myriocin reduces the expression of p-JNK, cleaved caspase-3, Bax, and cytochrome c, while increasing the expression of Bcl-2. Additionally, it reduces hepatic lipid accumulation in the hepatocyte of NAFLD rats, leading to a significantly improvement in liver inflammation and apoptosis in NAFLD rats [36]. In a separate study, rats supplemented with silicon in the diet showed a notable reduction in the number of apoptotic cells. The levels of apoptosis-related markers such as cytoplasmic cytochrome c, apoptosis-inducing factor AIF, caspase-3/9, and Bax/Bcl-2 ratio were significantly decreased, suggesting that silicon can reduce hepatocyte apoptosis, attenuate liver injury, and affect NASH development by modulating the mitochondrial intrinsic pathway [37]. In HFD-induced mice hepatocytes and PA-induced AML-12 cells, the researchers found that carnosol (CAR) treatment can maintain mitochondrial membrane potential and inhibit mitochondrial oxidative stress by specifically interacting with and upregulating the expression of the peroxiredoxin 3 (PRDX3), a mitochondrial H_2_O_2_ scavenger. This interaction improves mitochondrial function, facilitates ROS clearance, diminishes apoptosis, mitigates liver injury, and decelerates the progression of NAFLD [38]. Huang et al. conducted a study in which they found *Meretrix meretrix* oligopeptides (MMO), extracted from shellfish, was able to ameliorate mitochondrial dysfunction by decreasing mitochondrial membrane potential and maintaining intracellular ion levels in PA-induced human Chang liver cells, thus exerting an anti-apoptotic effect [39]. Selenoprotein M (SELENOM) acts as an upstream inhibitor of mitochondrial damage, which regulates the pro-mitochondrial autophagy factor Parkin via the AMPKα1–Mitofusin 2 (MFN2) pathway, which in turn regulates mitochondrial membrane potential and mitochondrial autophagy, and reduces mitochondria-associated apoptosis in hepatocytes. Overexpression of SELENOM reversed the upregulation of Bax, Cyt-c, caspase-3/9 and downregulation of Bcl-2 in PA-induced AML-12 cells, attenuating the level of apoptosis. In contrast, SELENOM deficiency increased HFD-induced apoptosis and lipid accumulation in mouse hepatocytes [40].

In addition, ERs-induced apoptosis is particularly important in the progression of NAFLD, as many drugs have been found to reduce the occurrence of apoptosis in hepatocytes by inhibiting ERs. Tanshinone IIA (Tan IIA) significantly inhibited the expression of ERK-related molecules GRP78, ATF6, and CHOP in HepG2 cells induced by PA, suppressed phosphorylation of eIF2α and reduced ERs-induced apoptosis [26]. Following 5 weeks of continuous administration of the hypoglycemic drug empagliflozin in HFD-fed mice, a decrease in NAFLD activity scores (NAS) was detected. The expression of ERs molecules was significantly downregulated, resulting in an increased Bcl-2/Bax ratio, inhibited caspase-8 cleavage, and a significant reduction in hepatocyte apoptosis. It shows that empagliflozin decelerates the progression of NAFLD by reducing ERs and inhibiting hepatocyte apoptosis [41]. MiR-149 can attenuate ERs-induced apoptosis by downregulating the ATF6 signaling pathway, thereby improving NAFLD [42]. Rabbits fed a high-cholesterol diet and supplemented with α-tocopherol exhibited inhibition of cholesterol-induced NASH development. This inhibition was achieved by reducing the JNK/c-Jun/inflammatory axis and JNK/CHOP/apoptotic signaling, which may contribute to resistance against the transformation of NAFLD into NASH [43]. The α-linolenic acid phytosterol ester PS-ALA inhibits ERs by regulating the AMPK signaling pathway, which in turn inhibits the activation of IRE1α/TRAF2/JNK signaling pathway, normalizes the Bcl-2 and Bax expression, and reduces ERs-induced hepatocyte apoptosis to achieve a preventive effect against NAFLD [44].

In addition, some other drugs or molecules inhibit hepatocyte apoptosis in other ways. In HFD-induced mice and PA-treated hepatocytes, knockdown of PIN2/TRF1-interacting telomerase inhibitor 1 (PinX1) could inhibit the expression of cleaved caspase-3 and cleaved poly ADP-ribose polymerase (PARP), one of the cleavage substrates of caspase, by upregulating the expression of telomerase reverse transcriptase (mTERT), exerting an anti-apoptotic effect. While PinX1 is a potential target for the treatment of NAFLD, the specific anti-apoptotic signaling pathway and molecular mechanism remain to be investigated [45]. In a study on NAFLD rat model, long non-coding RNA HULC (lncRNA HULC) could aggravate liver injury associated with NAFLD by activating the MAPK signaling pathway. In addition, the inhibition of HULC can reduce hepatocyte apoptosis and ameliorate liver fibrosis to a certain extent in NAFLD rats, and therefore, it will serve as a potential therapeutic target for NAFLD [46].

### Necroptosis

#### Overview of necroptosis

**Fig. 2:**
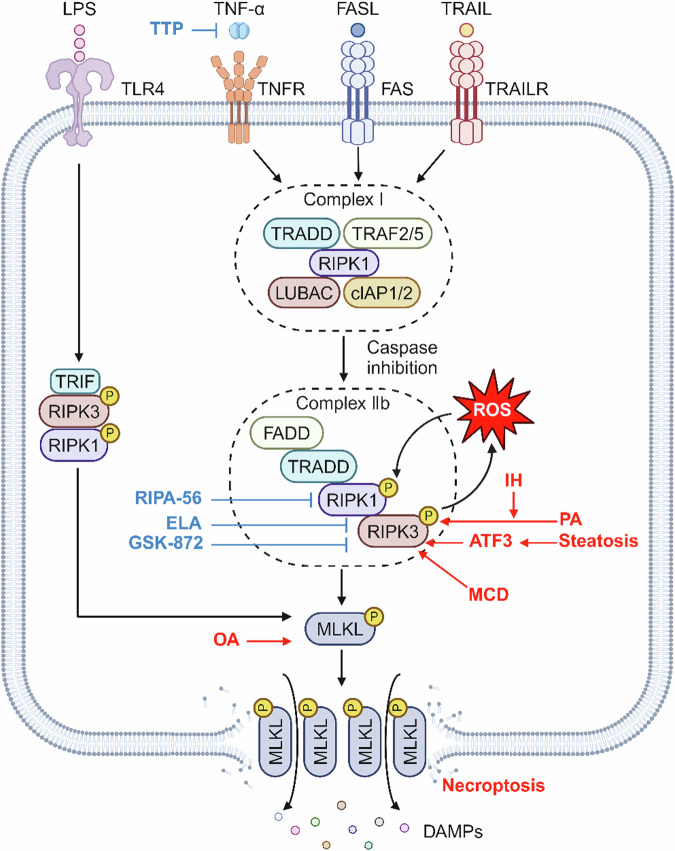
Mechanism of necroptosis in NAFLD. Various NAFLD triggering factors can lead to the upregulation of RIPK3, triggering RIPK3-dependent necroptosis, and drugs such as ELA and TTP can inhibit necroptosis and protect against NAFLD by acting on different targets of the necroptosis mechanism.

Necroptosis, a type of programmed inflammatory cell death, was first defined in 2005 by Degterev et al. [47] They found that the Fas/TNFR receptor family can activate a common non-apoptotic death pathway even in the absence of intracellular apoptotic signaling and it can be inhibited by specific chemical inhibitors of RIPK1, such as necrostatin-1 (Nec-1) and its modified analog Nec-1 (Fig. 2). As a result, this particular death pathway was named necroptosis. It is morphologically similar to necrosis and is characterized by increased cell volume, organelle swelling, and plasma membrane rupture [48]. The disruption of the plasma membrane leads to a rapid and extensive release of danger-associated molecular patterns (DAMPs), which subsequently trigger inflammation [49]. Although necroptosis is closely associated with apoptosis, it differs in that necroptosis is a specific type of caspase-independent cell death. Necroptosis shares the same death-inducing agents as apoptosis, including TNFα, first apoptotic signaling ligand (FasL), TRAIL, and toll-like receptor 3/4 (TLR3/4). In the context of TNF binding to TNFR1 and subsequent caspase-8 inhibition, the activation of receptor interacting protein 3 (RIPK3) triggers necroptosis [50, 51]. Active RIPK1 is recruited in an oligomeric complex including Fas-associated death domain (FADD) and caspase-8, activated caspase-8 can initiate apoptosis and also prevent necroptosis. Thus, the inhibition of caspase-8 promotes the transition from RIPK1-induced apoptosis (complex IIa) to necroptosis, where RIPK1 recruits and phosphorylates RIPK3, and activated RIPK3 further stimulates the activation of mixed lineage kinase domain-like (MLKL) through the way of phosphorylation. Phosphorylated MLKL translocates to the inner leaflet of the plasma membrane, disrupting cellular integrity and thus leading to necroptosis [52–54]. In addition, MtROS could promote necroptosis. Activated RIPK3 induces phosphorylation of MLKL and ROS production, while RIPK1 is the major target of MtROS, thus forming a positive feedback pathway for necroptosis [55].

#### Necroptosis in NAFLD

Necroptosis has been validated to be closely associated with NAFLD and NASH in animal models and human diseases. Expression of necroptosis-associated factors MLKL, RIPK1, and RIPK3 in the liver can be used to diagnose and assess NAFLD progression [56]. Epigenetic silencing of RIPK3 in mouse and human primary hepatocytes prevents necroptosis-mediated liver pathological damage induced by MLKL activation [57]. Both RIPK3 and MLKL expression were increased in liver tissues of NAFLD and NASH patients [58, 59]. Similar to those findings, steatohepatitis was exacerbated after 18 weeks of feeding mice with a high-fat choline-deficient (HFCD) diet, and RIPK3 and MLKL were sequestrated in the insoluble protein fraction of liver lysates from NASH mice, suggesting early events during steatohepatitis progression [58]. MLKL expression in liver tissue of NAFLD patients increases in correlation with the severity of associated pathological changes, such as steatosis, ballooning, and inflammation. Compared to control mice, MLKL-KO mice fed with the HFD exhibited reduced NAS, steatosis score, inflammation, and ballooning degeneration [60]. In the methionine choline-deficient (MCD) diet-fed NASH model, the primary role of caspase-8 is to prevent excessive activation of necroptosis and caspase-8-deficient mice develop large amounts of RIPK3 in hepatocytes, leading to severe liver injury and fibrosis. Conversely, mice lacking RIP3 significantly reduced liver injury, inflammation, and fibrosis induced by the MCD diet compared to wild-type mice, indicating an important role for RIPK3-dependent necroptosis in the progression of NAFLD [61]. In contrast to HFD-fed mice, the heme oxygenase-1 (HO-1) (ROS scavenger) expression and MLKL phosphorylation were increased in sucrose-containing HFD-fed mice, while both indicators were also increased in human NAFLD patients. Excess fructose can induce NAFLD progression by enhancing OA lipotoxicity through ROS production, leading to hepatocyte necroptosis, and its effects are ameliorated by treatment with the reactive oxygen scavenger NAC [62].

RIPK3 was found to be overexpressed in the white adipose tissue (WAT) of HFCD-fed obese mice as well as in visceral WAT of obese humans, and although RIPK3 mediates necroptosis, it primarily inhibits apoptosis, which suppresses inflammation and maintains tissue homeostasis [63]. Interestingly, RIPK3 has been identified as a key mediator of necroptosis that protects mice from HFD-induced liver injury, which contrasts with previous observation in mice on the MCD diet that RIPK3 promotes NAFLD progression. The absence of RIPK3 aggravates HFD-induced liver injury, promotes hepatic inflammation and hepatocyte apoptosis, and is associated with an early fibrotic response. These findings suggest that altered patterns of hepatocyte death can influence NAFLD disease progression, where the complex balance and interplay of different pathways of PCD may be affected by the metabolic state of the liver [61, 64].

Regarding the triggering mechanism of necroptosis in the progression of NAFLD, it is speculated that it may be related to the stimulation of necroptosis-related first activation signals according to the reports. In NASH patients as well as in animal models of NAFLD, the expression of both TNF-α and TNFR1 was found to be upregulated. TNF-α levels positively correlated to the severity of liver disease, and TNFR1 deficiency exerts a protective effect against hepatic steatosis and liver damage [17, 65, 66]. Furthermore, hepatocytes are heavily exposed to TLR4 ligands during NASH [67]. When hepatocytes are exposed to these stimulatory signals, the necroptosis signaling pathway is triggered, which in turn triggers liver injury. Indeed, these necroptosis-associated signaling molecules can also stimulate apoptosis, and both necroptosis and apoptosis may be activated concurrently in liver injury, and RIPK3 can convert apoptosis to necroptosis only when caspase-8 is inhibited [51]. Whereas, with increased hepatic steatosis, the transcription factor ATF3 also increases hepatocyte death by inducing RIPK3 expression and changing the TNFα-dependent cell death pattern from apoptosis to necroptosis [68]. In exploring obstructive sleep apnea hypopnea syndrome (OSAHS)-associated NAFLD, researchers found that intermittent hypoxia (IH) increased RIPK3 upregulation caused by PA treatment of LO2 cells, promoted RIPK3-dependent necroptosis, and increases hepatic oxidative stress, and inflammatory responses. It was also found that IH-exacerbated NAFLD was regulated through the RIPK3-dependent necroptosis NRF2/nuclear factor kappa-B (NF-κB) signaling pathway [69]. Overall, we have to acknowledge the crucial role of necroptosis in the progression of NAFLD.

#### Necroptosis-targeted therapies in NAFLD

Researchers found that the dual peroxisome proliferator-activated receptor alpha/delta agonist Elafibranor (ELA) exerts a hepatoprotective effect by reducing cleaved RIPK3 and cleaved caspase-3, which in turn reduced hepatocyte necroptosis and apoptosis, inhibited hepatic steatosis, inflammation, and fibrosis, resulting in significantly lowered NASs [70]. RIPA-56, a specific inhibitor of RIPK1, and GSK-872, a RIPK3 inhibitor, have been shown to inhibit the RIPK1/RIPK3/MLKL pathway-mediated necroptosis. In HFD-fed mice, the use of these inhibitors targeting RIPK1/RIPK3 significantly reduces all hepatic features associated with steatohepatitis, including inflammation, liver fibrosis, transaminase activity, and triglyceride levels. Similarly, the same effect was observed by pharmacological inhibition or knockdown of the downstream MLKL of RIPK [69, 71]. In a recent report, researchers found that metformin, a commonly used medication for type 2 diabetes, activates the tristetraprolin (TTP) via the AMPK–SIRT1 pathway in hepatocytes and Kupffer cells (KCs). TTP inhibits TNF-α production in KCs, thereby reducing hepatocyte necroptosis [72]. In addition, AS1842856 attenuates necroptosis in NAFLD mice by specifically inhibiting Forkhead box O (FOXO1), a key downstream factor of the insulin/IGF-1 signaling pathway. RIPK1, RIPK3, and phosphorylated MLKL were significantly downregulated in AML-12 cells treated with AS1842856, thereby mitigating necroptosis marker protein upregulation due to PA treatment. FOXO1 is involved in the regulation of glucose metabolism, however, its role in NAFLD and its relationship with necroptosis and molecular mechanisms remain to be investigated [73].

### Pyroptosis

#### Overview of pyroptosis

**Fig. 3:**
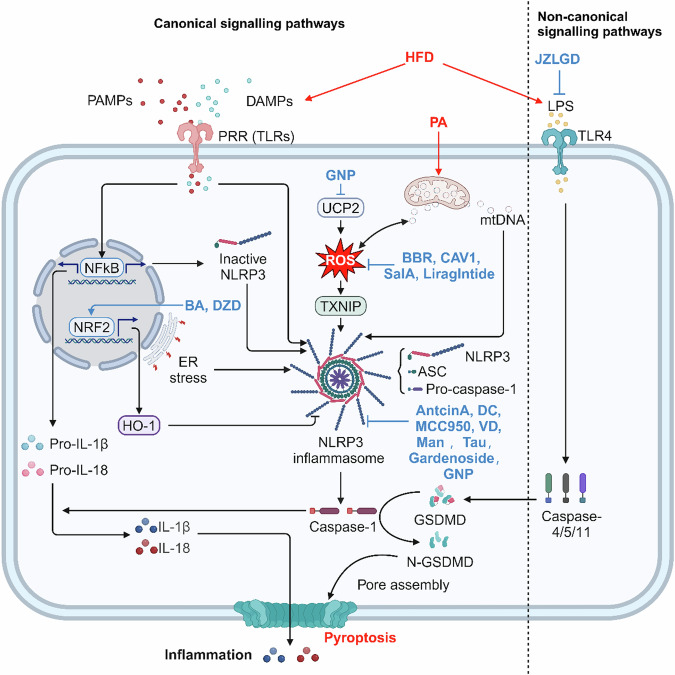
Mechanism of pyroptosis in NAFLD. Various NAFLD triggering factors can activate canonical and non-canonical signaling pathways-induced hepatocyte death through different mechanisms. Antcin A, Dieckol, and others can inhibit pyroptosis and prevent NAFLD by acting on different targets of the pyroptosis mechanism.

Pyroptosis was initially observed in 1992 by Zychlinsky et al. [74] in *Shigella fowleri*-infected macrophages and was defined as a caspase-1-dependent mode of pro-inflammatory PCD that is essential for the control of microbial infections. Pyroptosis occurs mainly during intracellular pathogen infection and is characterized by activation of the inflammasome, caspases-dependent formation of membrane pores, rupture of cell membrane swelling, and release of pro-inflammatory cytokines, such as IL-1β, IL-18, and cellular contents. These events collectively trigger subsequent immune responses to infection [75] (Fig. 3).

Pyroptosis is activated by canonical and non-canonical signaling pathways [76]. Inflammasome activation, known as the canonical signaling pathways, involves two steps. First, the binding of danger associated molecular patterns (DAMPs), pathogen associated molecular pattern (PAMPs), or cytokines to TLRs triggers the activation of NF-κB signaling pathway, resulting in an upregulation in the expression of pro-IL-1β, pro-IL-18, and NOD-like receptor thermal protein domain associated protein 3 (NLRP3). Second, the inflammasome begin to assemble and form. DAMPs caused by environmental factors and cellular damage stimulate inflammasome sensor proteins such as NOD-like receptor proteins (NLRP1, NLRP3, NLRC4, NLRP6), absent in melanoma 2 (AIM2)-like receptors (ALRs), or pyrin families. Typical inflammasome components include the junctional protein named apoptosis-associated speck-like protein containing a CARD (ASC) and the inactive zymogen pro-caspase-1 [77, 78]. NLRP3 inflammasome activates caspase-1 to cleaves pro-IL-1β and pro-IL-18 into the active forms IL-1β and IL-18, meanwhile, cleaves the amino-terminal fragment of the key protein gasdermin D (GSDMD) to form N-GSDMD, which oligomerizes and forms membrane pore formation leading to pyroptosis and subsequent inflammatory response. In the non-canonical activation pathway of NLRP3, the presence of intracellular bacterial lipopolysaccharide (LPS) is detected by caspase-4/5/11, leading to proteolytic activation of GSDMD, which in turn leads to pyroptosis in gram-negative infections [79–81].

#### Pyroptosis in NAFLD

Previous studies have provided evidence supporting the involvement of pyroptosis, induced by the activation of inflammasomes, in the pathogenic mechanism of NAFLD. Hepatic mRNA levels of NLRP3, pro-caspase-1, IL-1β, and IL-18 were remarkably higher in NAFLD patients than in healthy individuals, and exhibited a strong correlation with lobular inflammation, hepatocyte ballooning, and NASs [82]. The suppression of key signaling molecules involved in the canonical signaling pathways of pyroptosis, (e.g., NLRP3, caspase-1, IL-1β, and GSDMD), was able to attenuate pathological changes such as hepatic steatosis, inflammation, and early fibrogenesis in animal models of NAFLD/NASH. This suggests the crucial role of pyroptosis in the pathogenesis of NAFLD/NASH [83–87]. During the development of NASH, activation of NLRP3 inflammasome mediates caspase-1 activation in bone marrow-derived KCs, which then release pro-inflammatory signals that can activate hepatic stellate cells (HSCs) responsible for collagen deposition and fibrosis in the liver [84]. Additionally, the internalization of NLRP3 inflammasome particles by HSCs induces their activation, resulting in increased secretion of IL-1β and expression of α-smooth muscle actin (α-SMA) [88, 89]. Over time, this process gradually leads to severe liver injury and liver fibrosis.

The first step of risk signals for inflammasome activation includes mainly DAMPs and PAMPs, leading to the activation and upregulation of NLRP3, pro-IL-1β, and pro-IL-18. The second step signals are dependent on cellular stress in the process of NAFLD/NASH including MtROS, mtDNA, ERs, and impaired mitochondrial phagocytosis, etc. [89]. Saturated FAs represent an endogenous hazards that upregulate the inflammasome in NASH in the form of a first hit and induce sensitization of hepatocytes to a second hit of LPS, resulting in the release of IL-1β [90]. Interestingly, PA activates NLRP3 inflammasome in mouse KCs by decreasing the mitochondrial membrane potential and inducing the release of mtDNA from mitochondria into the cytoplasm to form mtDNA–NLRP3 inflammasome complexes [91]. Apart from the activation of NLRP3 inflammasome in NAFLD, mitochondrial damage and mtDNA release can also trigger the activation of AIM2 inflammasome [92]. The activation of NLRC4 inflammasome is modulated by TNF-α. Increased TNF-α promotes the activation of NLRC4 inflammasome in the liver, which improves the production of IL-18 and IL-1β, triggers hepatocyte pyroptosis, and accelerates NAFLD progress [93].

Drummer et al. [94] reported that HFD-feeding increased the production of endotoxin LPS by gram-negative bacteria in the intestinal microbiota of mice. The elevated levels of LPS in circulation and intracellularly activates caspase-11 and initiates caspase-11-dependent non-canonical signaling pathways, eventually leading to pyroptosis. Additionally, PA produced by lipolysis in HFD-fed mice also activates caspase-11 and GSDMD cleavage [94]. Furthermore, the involvement of caspase-11 in mediating hepatocyte pyroptosis was verified in an MCD-induced NASH mouse model. The deficiency of caspase-11 significantly reduced liver injury and inflammation in mice, downregulated the activation of GSDMD and IL-1β, and the expression of fibrillation-related factors (TGF-β, α-SMA, and alpha-1 type I collagen) was also reduced, revealing a critical role for caspase-11 in NASH [95].

#### Pyroptosis-targeted therapies in NAFLD

Recent studies have shown that Exenatide treatment can effectively reduce the expression of NLRP3, caspase-1, and IL-1β in OA/LPS-induced HepG2 cells and MCD-induced db/db mouse livers, which in turn inhibits the pyroptosis signaling pathway to alleviate NASH [96]. Similarly, the herbal formulation Jinlida granule has been found to reduce the expression of NLRP3, caspase-1, IL-1β, and IL-18 in HFD-induced mouse livers as well as FFA-induced HepG2 cells, demonstrating its potential to alleviate liver injury and exert a protective effect against NAFLD by reducing hepatocyte pyroptosis [97]. However, the exact mechanism of their action remains to be studied.

Antcin A, a small triterpene molecule, indirectly inhibits pyroptosis by targeting the assembly and activation of NLRP3 inflammasome [98]. Similar to Antcin A, compounds such as Dieckol (DK), MCC950 (a specific NLRP3 inhibitor), and VD also reduce pyroptosis by targeting NLRP3 inflammasome, thereby attenuating the accumulation of triglycerides and FFA, improving liver injury, and reducing NAFLD scores [99–101]. Mangiferin (Man), on the one hand, downregulated the expression of NLRP3 inflammasome-related proteins to attenuate pyroptosis and inflammatory effects. On the other hand, it upregulates p-AMPKα levels, regulating glucolipid metabolism and ameliorating liver injury, insulin resistance, glucose tolerance, lipid accumulation, and inflammation in NAFLD mice [102]. In an As_2_O_3_-induced NASH model, researchers found that taurine (Tau) inhibits pyroptosis and attenuates hepatic inflammation through inhibition of the cathepsin B (CTSB)–NLRP3 inflammatory vesicle pathway [103]. Gardenoside, a natural botanical ingredient, ameliorates lipid accumulation and liver fibrosis by reducing the expression of pyroptosis-associated protein. The knockdown of CCCTC binding factor (CTCF) or dipeptidyl peptidase-4 (DPP4) in HFD-fed mice and PA + LPS-treated AML-12 cells yields similar effects to Gardenoside treatment, while CTCF overexpression counteracted this alteration. These findings suggest that Gardenoside inhibits via the CTCF/DPP4 signaling pathway [104]. Genipin (GNP), acting as an inhibitor of uncoupling protein-2 (UCP2), effectively reversed HFD-induced liver injury and inhibited NLRP3 inflammasome activation. Notably, its inhibitory effect on inflammasome was verified to be related to UCP2–ROS signaling [105]. Besides, lncRNA growth-arrest specific transcript 5 (GAS5) also inhibits NLRP3 inflammasome-mediated hepatocyte pyroptosis via binding miR-28a-5p in NAFLD [106].

Caveolin-1 (CAV1) may inhibit NLRP3-mediated pyroptosis through the ROS/thioredoxin-interacting protein (TXNIP) pathway. In detail, CAV1 scavenges ROS, preventing the separation of TXNIP from thioredoxin and its binding to NLRP3, thus inhibiting the assembly and activation of the NLRP3 inflammasome and attenuating liver injury [107]. Apart from CAV1, additional chemical medicines, such as Berberine (BBR) and Salvianolic acid A (SalA), have been found to inhibit pyroptosis during the NAFLD process by modulating TXNIP-induced NLRP3 inflammasome activation [108, 109]. Additionally, Liraglutide has shown promise in human and experimental studies for the prevention of NASH by inhibiting NLRP3 inflammasome activation and pyroptosis in PA + LPS-treated hepatocytes. This is achieved through the reduction of lipid accumulation, maintenance of mitochondrial function, and decrease in ROS production [110].

It was well known that, under physiological conditions, the NRF2/HO-1 axis is essential for resistance to oxidative stress, and upregulation of this axis significantly suppresses the expression of NLRP3, caspase-1, and ASC, inhibits inflammasome formation, blocks GSDMD activation, and prevents the release of IL-1β and IL-18. In exploring the mechanisms underlying the lipid-lowering and anti-inflammatory effects of Baicalin (BA), it was discovered that BA reduced FFA-induced HepG2 cells and HFD-induced mice hepatocyte pyroptosis via the NRF2/HO-1/NLRP3 pathway. However, the regulation of NRF2 by BA needs to be further investigated [111, 112]. Indeed, Danshen zexie decoction, like BA, protects against HFD-induced inflammation and liver injury by upregulating of this axis [113].

In studies of non-canonical signaling pathways, it has been found that Jiangzhi Ligan Decoction (JZLGD) ameliorated steatosis, inflammation, and pyroptosis in HFD-fed rats not only by inhibiting the NLRP3/caspase-1/GSDMD-mediated typical pyroptosis pathway but also by decreasing the levels of LPS in the hepatic portal vein of NAFLD rats, which prevented caspase-11 and GSDMD activation, inhibited the LPS/caspase-11/GSDMD-mediated non-canonical signaling pathways, and alleviated NALFD [114].

### Ferroptosis

#### Overview of ferroptosis

**Fig. 4:**
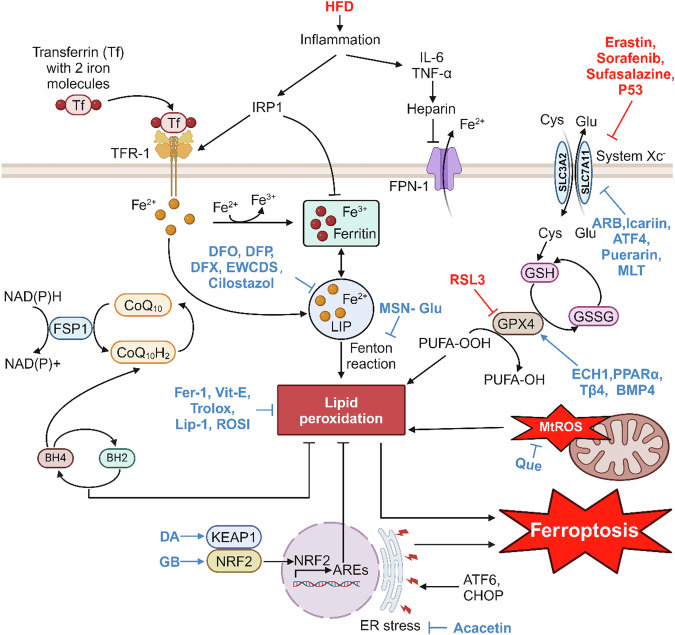
Mechanism of ferroptosis in NAFLD. Iron metabolism imbalance in the liver during NAFLD development triggers ferroptosis, leading to lipid peroxidation and accelerated NAFLD progression, and drugs such as EWCDS and BMP4 can inhibit ferroptosis and protect against NAFLD by acting on different targets of the ferroptosis mechanism.

Ferroptosis is a novel mode of PCD that is iron-dependent and distinct from apoptosis, necroptosis, and pyroptosis. Early in 2012, it was first described by Dixon et al. [115] and the central events of ferroptosis involve intracellular iron overload, excessive accumulation of lipid ROS, and lipid peroxidation. It differs morphologically, physiologically, and genetically from apoptosis, necroptosis, and pyroptosis, making it a unique form of cell death (Fig. 4). Morphologically, it is characterized by cell membrane rupture and effacement, a decline or even vanishment of mitochondrial cristae, mitochondrial atrophy, and normal nuclear morphology but it lacks of chromatin condensation [115].

Ferroptosis is intertwined in three aspects: amino acid and lipid metabolism, regulation of ROS, and iron regulation. As we know, the key components of the cell membrane are phospholipids (PLs), and when PUFAs are part of the membrane PLs, they become substrates for the peroxidation of cellular ferroptosis. The ferroptosis activator Erastin can act directly on systemic Xc^−^, the reverse transporter of cystine/glutamate in the cell membrane, to inhibit amino acid uptake, which in turn affects glutathione (GSH) synthesis, causing a decrease in glutathione peroxidase 4 (GPX4) activity [115, 116]. Under basal conditions, GPX4 converts GSH to oxidized glutathione (GSSG) and reduces cytotoxic lipid peroxides (L-OOH) to the corresponding alcohols (L-OH). In response to GPX4 inhibition, cellular antioxidant capacity is rapidly reduced, which in turn leads to the accumulation of intracellular lipid ROS, oxidative damage, and ultimately ferroptosis. Unlike the canonical ferroptosis pathway due to GPX4 inactivation, intracellular Fe^2+^ accumulation, which can react with peroxides in the Fenton reaction, generates excess hydroxyl radicals, induces lipid peroxidation, and drives cellular ferroptosis [117, 118]. Systemic Xc^−^ inhibitors (e.g., Sorafenib, Sulfasalazine, and Erastin) are considered as class I ferroptosis inducers, whereas directly GPX4 inhibitors such as RAS selective lethality protein 3 (RSL3), are referred to as class II inducers [119–121]. Some transcription factors regulate ferroptosis-related genes in response to oxidative stress, for example, p53 decreases cystine uptake by inhibiting the expression of Solute carrier family 7 member 11 (SLC7A11), one of the components of the Xc^−^ system, thereby increasing cellular susceptibility to ferroptosis [122]. And ferroptosis induced due to GPX4 deficiency can be inhibited by ferroptosis suppressor protein 1 (FSP1) through the NAD(P)H–FSP1–CoQ10 axis. This is a signaling pathway parallel to the Cyst(e)ine–GSH–GPX4 axis, which plays an important role in the inhibition of lipid peroxidation and ferroptosis [123]. In addition, there exists another pathway that inhibits ferroptosis, the GCH1–BH4–DHFR axis. Tetrahydrobiopterin (BH4), a downstream factor of GTP cyclohydrolase-1(GCH1), on the one hand, inhibits ferroptosis by promoting the synthesis of CoQ10 and, on the other hand, inhibits lipid peroxidation as an antioxidant, which, in turn, inhibits ferroptosis [124, 125].

#### Ferroptosis in NAFLD

Numerous studies have demonstrated the presence of iron overload in the liver tissue of NAFLD patients, that excess free iron can generate hydroxyl radicals and ROS via the Fenton reaction, promoting lipid peroxidation, and that redox imbalance is a central pathogenic mechanism of NAFLD and its progression [118, 126]. Maintaining a balance of iron metabolism in the body prevents obesity and increases insulin sensitivity, both of which are typical of NAFLD patients [127, 128]. It is estimated that one-third of patients with NAFLD present with methemoglobinemia, mainly with mild hepatic iron deposition (50–150 μmol/g) [128]. Kowdley et al. examined the connection between increased human serum ferritin (SF) and NAFLD severity, and they found that SF > 1.5 × upper limit of normal (ULN) independently predicted the development of advanced liver fibrosis in patients with NAFLD and that elevated SF was independently linked with elevated NAS even in patients without hepatic iron deposition correlated [129]. Indeed, iron metabolism disorders and ferroptosis occur in the early stages of the NAFLD process. On the one hand, in the liver of HFD-fed rats, the activity of iron regulatory protein 1 (IRP1) is increased, and it affects iron uptake and storage in cells by transcriptionally regulating the expression of ferritin, transferrin receptor-1 (TfR1) and iron export protein ferroportin (FPN-1). On the other hand, HFD induced in a time-dependent manner the expression of hepatic pro-inflammatory cytokine IL-6 and transcription factor C/EBPα, both of which promoted the expression of ferroportin, and, in turn, the downregulation of FPN-1, ultimately leading to an increase in iron content (HIC) in hepatocytes. These findings suggest a strong link between an imbalance of iron metabolism, inflammatory damage, and disease progression [130].

In addition to iron overload, lipid peroxidation is an essential process for the development of ferroptosis [131]. Loguercio et al. [132] observed that more than 90% of patients with NAFLD exhibited elevated levels of lipid peroxidation markers (malondialdehyde [MDA] and 4-hydroxynonenal [4-HNE]), and in particular, patients with NASH had significantly higher MDA and 4-HNE than patients with steatosis [132]. Qi et al. [133] demonstrated that in the MCD-induced NASH model, treatment with the GPX4 activator sodium selenite, the iron chelator desferrioxamine (DFO), and the ferroptosis inhibitor Liproxstatin-1(Lip-1) inhibited the ferroptosis, suppressed inflammatory response, and alleviated NASH, while conversely RSL3 (a ferroptosis inducer) downregulates GPX4 levels and exacerbates the severity of NASH [133]. Likewise, the presence of ferroptosis was confirmed in an arsenic-induced NASH model by detecting the expression of ferroptosis-related proteins as well as mitochondrial morphology [134]. Tsurusaki et al. [135] found that necroptosis precedes apoptosis in the process of NAFLD through a choline-deficient, ethionine-supplemented (CDE) diet model. However, after administration of necroptosis and ferroptosis inhibitors on top of the CDE diet, it was found that ferroptosis inhibitors almost completely reversed hepatocyte death, whereas necroptosis inhibitors failed to block the onset of cell death, suggesting that hepatocyte ferroptosis may be one of the initial modes of cell death at the onset of steatohepatitis and may be a trigger for the initial inflammation of steatohepatitis [135]. In summary, these studies prove that ferroptosis is involved in NAFLD and accelerates its developmental process.

#### Ferroptosis-targeted therapies in NAFLD

Therefore, inhibition of ferroptosis is a promising therapeutic strategy for NAFLD, and an increasing number of drugs targeting various pathways involved in the occurrence of ferroptosis have been identified, which can effectively target different ferroptosis-related factors. Consequently, these drugs inhibit ferroptosis, reduce lipid peroxidation and oxidative stress in cells, and slow down the progression of NAFLD. Notably, some ferroptosis inhibitors such as iron chelators, Fer-1, Lip-1, ROSI (ACSL4 inhibitor), and Trolox (antioxidant vitamin E analog) have been demonstrated to significantly inhibit ferroptosis in NAFLD animal models induced by arsenic, CDE-fed, MCD-fed, HFD-fed, and so on, and attenuate liver inflammation reactions, lipid accumulation, and liver injury [134–138].

In addition to some traditional iron chelators including DFO, deferiprone (DFP), and deferasirox (DFX), a novel iron nano-chelator fluorescent protein-based carbon dots (EWCDs) can effectively reduce ROS production, inhibit apoptosis, and alleviate inflammation, making EWCDs a potential treatment for zebrafish iron overload-induced NAFLD. What’s more, EWCDs can inhibit hepatocyte apoptosis and reduce inflammation by attenuating ERs [139]. Similarly, Insulin-like growth factor binding protein 7 (IGFBP7) deletion in the zebrafish NAFLD model significantly increased the expression of the nuclear receptor coactivator 4 (NCOA4), increased NCOA4-mediated ferritin autophagy (ferritinophagy), maintained intracellular iron homeostasis, and reduced the occurrence of hepatocyte ferroptosis [140]. Cilostazol inhibits ectopic erythrocyte phagocytosis by hepatic inhibition of increased platelet and erythrocyte accumulation, which in turn attenuates hepatic iron overload and prevents ferroptosis [141]. The above drugs reduce ferroptosis by directly or indirectly reducing the accumulation of Fe^2+^ in cells.

Accordingly, there are many other drugs that ultimately target Systemic Xc^−^ in various ways, thereby reducing cystine uptake and inhibiting ferroptosis. The natural antioxidant arbutin (ARB) is able to target demethylase fat mass and obesity-related protein to promote methylation of the SLC7A11 gene, one of the components of Xc^−^, improving HFD-induced NAFLD in vivo and in vitro [142]. Icariin, a flavonoid abundant in the herb Epimedium, ameliorated MCD diet-induced lipid peroxidation, restored mitochondrial structure in the livers of NASH mice, and exerted hepatoprotective effects through the activation of Nrf2, which in turn significantly elevated the protein levels of GPX4 and a cystine/glutamate transporter (xCT) [143]. Activating transcription factor 4 (ATF4) was also able to inhibit ferroptosis and improve liver function by increasing the expression of SLC7A11 [144]. Similarly, in HFD combined with streptozotocin (STZ) injected mice and PA-treated AML-12 cells, researchers found that Puerarin treatment was able to effectively inhibit ferroptosis and reduce lipid peroxidation by activating the SIRT1/NRF2 pathway and increasing the expression of HO-1, SLC7A11, and GPX4 proteins [145]. HO-1 is the major and rate-limiting enzyme of heme metabolism, which catalyzes the catabolism of heme to biliverdin, free iron, and carbon monoxide, and HO-1 deficiency exacerbates ROS accumulation, lipid peroxidation, and iron overload in hepatocytes [146]. In FFAs-treated HepG2 cells and HFD-induced NAFLD mice, researchers found that Melatonin treatment increased NRF2 and HO-1 levels to protect cells from oxidative environment, and also upregulated the expression of GPX4 and SLC7A11 to inhibit ferroptosis [147].

In addition, in the antioxidant signaling pathway Keap1/NRF2/ARE, Gao et al. found that a substance called dehydroabietic acid binds to Keap1, activates NRF2-ARE, induces downstream gene expression, which in turn upregulates GSH, GPX4, inhibits ROS accumulation and lipid peroxidation, suppresses ferroptosis and NAFLD [148]. Yang et al. [149] found that Ginkgolide B activated the NRF2 signaling pathway by promoting NRF2 translocation to the nucleus and upregulated GPX4, which in turn inhibited ferroptosis in PA/OA-induced HepG2 cells and HFD-induced mice hepatocytes. In addition, there are a number of genes and small molecule compounds that have been shown to upregulate GPX4 through other pathways. For instance, enoyl coenzyme A hydratase 1 (ECH1) can reduce ferroptosis by upregulating GPX4 on the one hand, and reduce apoptosis by inhibiting ERK on the other hand, thus improving NAFLD [150]. In addition, PPARα inhibits ferroptosis by promoting GPX4 expression and inhibiting plasma transferrin (TRF) expression. The family of PPARs regulates energy metabolism, of which PPARα is mainly expressed in the liver and can directly induce GPX4 expression [151]. Shu et al. [152] found that time-restricted feeding suppressed the expression of the circadian gene Per2, and that specific knockdown of Per2 in hepatocytes promoted the expression of PPARα, thereby reducing lipid peroxidation levels, inhibiting the ferroptosis-related genes expression, improving mitochondrial morphology, and ultimately effectively mitigating NASH. Another novel strategy and treatment target for NAFLD identified by Zhu et al. [153] is thymosin beta 4 (Tβ4), which can exert a protective effect against NAFLD by inhibiting ferroptosis in PA-induced LO2 cells and HFD-induced rat hepatocytes through upregulation of GPX4 expression [153]. Similarly the protective effect of bone morphogenetic protein 4 (BMP4) on NAFLD is mediated by regulating GPX4 expression through a non-transcriptional mechanism. The overexpression of BMP4 in LO2 cells and HepG2 cells can upregulate the expression of GPX4, reduce ROS and MDA levels, mitigate ferroptosis, and reduce liver injury [154].

There are also drugs that inhibit ferroptosis through other pathways that also exert a protective effect against NAFLD. Quercetin can attenuate lipid droplet accumulation and lipid peroxidation, ameliorate liver injury, and alleviate NAFLD by inhibiting MtROS-mediated ferroptosis [155]. Jiang et al. [156] demonstrated that ERs are an upstream signal for ferroptosis in NAFLD, and proposed a drug, the natural flavonoid Acacetin, which inhibits ferroptosis and reduces hepatic lipid accumulation by inhibiting ERs. Liraglutide, an agonist of GLP-1R, can increase acetyl-CoA carboxylase (ACC) phosphorylation through activation of AMPK, which in turn attenuates Erastin- or RSL3-induced cellular ferroptosis in the presence of high glucose, suggesting that Liraglutide ameliorates T2DM-associated NAFLD by inhibiting ferroptosis through activation of AMPK/ACC signaling [157].

In addition to these traditional iron death drugs, Zhao et al. developed for the first time a novel nanodrug: the *N*-(3-triethoxysilylpropyl)gluconamide (Glu) modified magnesium silicide (Mg_2_Si) nanosheets (MSN-Glu), which antagonizes the free heme-induced Fenton reaction by targeting H_2_ delivery to hepatocytes, reduces oxidative stress, lipid peroxidation, and subsequent ferroptosis, and significantly improves hepatic metabolic function in NAFLD mice [158].

## Cell death in different stages of NAFLD

NAFLD refers to a spectrum of liver damage ranging from NAFL to NASH and advanced liver fibrosis/cirrhosis. Chronic lipid accumulation in the liver causes inflammation, converts NAFL to NASH, and promotes hepatic fibrosis, with advanced progression to liver cirrhosis and hepatocellular carcinoma.

In recent years, a large number of studies have demonstrated that different types of cell death (including apoptosis, pyroptosis, necroptosis, and ferroptosis) occur concurrently in the progression of NAFL to NASH and that their occurrence correlates with the severity of the disease. Compared with patients with steatosis alone (NAFL), patients with NASH have a significantly increased level of apoptosis [12], as well as pyroptosis with elevated expression of caspase-1 [159] and necroptosis with increased expression of RIP3 [58]. In addition, the activation of HSC have been wildly reported to function in the progression of NASH to cirrhosis [160]. Notably, all the four types of cell deaths were reported to accelerate NASH by promoting HSC activation. Gaul et al. demonstrate that primary mouse and human hepatocytes can undergo pyroptosis with subsequent release of NLRP3 inflammasome proteins, which activates HSCs with increased secretion of IL-1β and α-SMA expression [159]. Human HSCs incubated with conditioned medium from steatotic hepatocytes show fibrogenic activation and enhanced resistance to apoptosis, suggesting that apoptotic hepatocytes could stimulate HSCs in patients with NASH [161]. Moreover, loss of MLKL, the terminal effector in necroptosis pathway, ameliorates liver fibrosis by inhibiting hepatocyte necroptosis and HSC activation [162]. As to ferroptosis, it plays as a “double-edged sword” in liver fibrosis. On the one hand, hepatocyte ferroptosis can aggravate liver fibrogenesis by activating HSCs [163], while on the other hand, treatment with Erastin in mice or Sorafenib monotherapy in advanced fibrotic patients with hepatocellular carcinoma could alleviate liver fibrosis by inducing HSC ferroptosis [164, 165]. Taken together, in the stage from NAFL to NASH, levels of cell death could be used as indicators of the pathogenesis with detection of the death-related markers, whereas in the stage from NASH to cirrhosis, cell death pathways are effective therapeutic targets in blockage of HSC activation.

## The cross-talk among different cell deaths in NAFLD

**Fig. 5:**
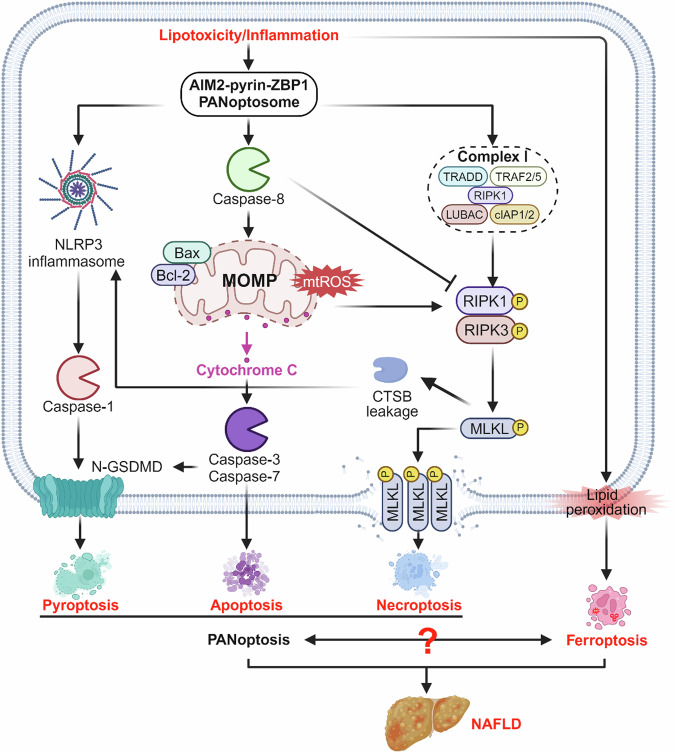
Cross-talk among different cell deaths. The three modes of death, including apoptosis, pyroptosis, and necroptosis, which coexist in NAFLD and interact with each other, are called PANoptosis. However, the interaction between PANoptosis and ferroptosis is not yet known.

Given that the different types of PCD, including apoptosis, necroptosis, pyroptosis, and ferroptosis, can coexist during the pathogenesis of NAFLD, there might be extensive interactions between the different modes of cell death. PANoptosis, including apoptosis, pyroptosis, and necroptosis, is newly proposed as a coordinated cell death pathway in recent years, which has been reported to play critical roles in the pathogenesis of NAFLD [166, 167]. During infections, AIM2 regulated the innate immune sensors Pyrin and ZBP1 to drive PANoptosis and provide host protection [168]. Si-Wu-Tang, a traditional Chinese herbal prescription, was reported to protect against hepatitis-mediated hepatocyte PANoptosis through inhibiting mtDNA release-mediated ZBP1 activation [167]. In monocytes and macrophages, caspase-1 activates caspases-3/7 and induces apoptosis in the absence of GSDMD while caspases-3/7 specifically block pyroptosis by cleaving GSDMD during apoptosis, which is called “bidirectional crosstalk” [169]. Whereas in the progression of NAFL to NASH, ghrelin shows beneficial effects via inhibition of TNF-α-induced hepatocyte apoptosis and pyroptosis [170]. Sodium sulfite, a biological derivative of the air pollutant sulfur dioxide, could trigger hepatic apoptosis, necroptosis, and pyroptosis in mice and hepatocye cell line [171]. Interestingly, in a 3-week non-alcoholic steatohepatitis mouse model, Elafibranor showed strong benefits on NASH with improvement in hepatic inflammation, necroptosis, and apoptosis, but did not alter pyroptosis [172]. Tβ4, a multifunctional polypeptide, improved PA-induced LO2 damage through inhibition of apoptosis and ferroptosis. Furthermore, GPX4 knockdown attenuated therapeutic effect of Tβ4 on rat liver and LO2 cells with upregulation of apoptosis-related Bcl-2, Bax, and caspase-3 [153]. Besides, in addition to blocking the ferroptosis markers ACSL4 and ALOX15, ferroptosis inhibitor liproxstatin-1 also inhibited hepatic apoptosis, pyroptosis, and necroptosis by inhibiting cleavages of PANoptosis-related caspase-8 and caspase-6 in MAFLD mouse liver [173]. Taken together, more and more evidence regarding the interactions among apoptosis, pyroptosis and necroptosis and the relationship between panoptosis and ferroptosis is provided in pathogenesis of NAFLD interactions. However, although the ferroptosis and panoptosis are common targets of drugs, the underlying molecular mechanism is yet unknown (Fig. 5).

## Limitations

There exist several limitations, as different cell death subroutines display distinct feature, while sharing many similar characteristics with considerable overlap and cross-talk. Though it is recognized that various types of cell death may collectively contribute to the onset and progression of NAFLD, the specific dominant role of each type and the potential synergy resulting from the simultaneous activation of multiple pathways in hepatocytes remain elusive. To date, certain cell death modes, such as cuproptosis [174], remain poorly understood, and no literature has been found on the direct association with NAFLD.

## Conclusions and perspectives

This article reviews the molecular mechanisms of four different forms of cell death, demonstrates the existence of multiple forms of hepatocyte death in NAFLD and the acceleration of NAFLD progression by these forms of cell death in in vitro and in vivo experiments, and summarizes the therapeutic agents or potential therapeutic targets for the different forms of cell death in the existing studies. However, our understanding of the association between different forms of cell death and NAFLD is incomplete, and the pathophysiologic mechanisms are not yet clearly elucidated. Current studies have not yet been able to answer the question of the interrelationships and influences of the various forms of cell death in the disease, as well as the sequence and predominance of the various forms of cell death at different stages of the disease progression. Future research needs to focus on understanding the linkages between different forms of cell death pathways and their possible roles in NAFLD to help us better understand the complex mechanisms of the disease and to find more effective therapeutic agents that target cell death.
